# 

**Published:** 1846-05

**Authors:** 


### CONTENTS

OF NO. V.

### ORIGINAL COMMUNICATIONS.

### ESSAYS AND CASES.

ART. I.—An Essay on Diet in Health and Disease. By JOHN DAWSON, M.D., of Jamestown, Ohio. . 369

ART. II.—Remarks on Cataract; with a Case. By WM. M. BOLING, M.D., of Montgomery, Ala. - - 387

ART. III.—Medical and Obstetrical Cases. By FRANCIS H. GORDON, M.D., late President of Clinton College, Tennessee. - - - - - - - - - 390

ART. IV.—A Case of Alarming Hemorrhage from the Bowels in a little girl, caused by the detachment of a Polypus. By Dr. M. H. FEE, of Leatherwood, Ia. - 395

ART. V.—A Case of Purpura Hemorrhagica. By W. D. HARRIS, M.D., of Utica, Miss. - - - - 397

### REVIEWS.

ART. VI.--Animal Chemistry with reference to the Physiology and Pathology of Man. By J. FRANZ SIMON, Fellow of the Society for the advancement of Physiological Chemistry at Berlin, &c. &c. Translated by THOS. E. DAY, M.A. & L.M., Cantab., Licentiate of the Royal College of Physicians. - - - - 401

ART. VII.—Clinical Lectures on Surgery, delivered at St. George's Hospital. By SIR BENJAMIN C. BRODIE, Bart., V.P.R S., Sergeant Surgeon to the Queen; Surgeon in Ordinary to his Royal Highness Prince Albert, &c. &c. &c. - - - - - - - - 409

ART. VIII.—A Dictionary of Practical Medicine, comprising General Pathology, the Nature and Treatment of Diseases, Morbid Structures, &c. By JAS. COPLAND, M.D.,F.R.S. Edited, with additions, by CHAS. A. LEE, M.D. - - - - - - - - 414
ART. IX.—A Practical Treatise on the Diseases of Children. By JAMES STEWART, M.D., A.M., Fellow of the College of Physicians and Surgeons, one of the Consulting Physicians of the Northern Dispensary of the city of New York; late Physician to the Orphan Asylum, &c. &c. 3d edition, revised and enlarged. . 416

SELECTIONS FROM AMERICAN AND FOREIGN JOURNALS.

Report on the Progress of Forensic Medicine. - - - 417
Use of Cold Water in Erysipelas. - - - - - - 444
Influence of Menstruation upon the Exanthemata. - - 445
Intermittent character of certain diseases of the Nervous System. 446
Issues on the Scalp in Chronic Cerebral Diseases. - - 447
Syphilis. - - - - - - - - - - - 448

ORIGINAL INTELLIGENCE.

Pennsylvania Hospital. - - - - - - - - 449
Franklin Medical College of Philadelphia. - - - 452
Medical College of Memphis. - - - - - - 453
University of Maryland. - - - - - - - 453
The Medical Profession of the United States. - - - 454
A Correction. - - - - - - - - - - 456
The National Convention. - - - - - - - 457
Voyage of a Naturalist. - - - - - - - - 457
*Facts from Correspondents.*
A Case of Ileus relieved by Tobacco. - - . 458
A Case of Hemoptysis and Menorrhagia. - - 459
Convulsions from Disentery and Intermittent Fever. 460
Tincture of Iodine in the treatment of Porrigo. - 460

